# FCET2EC (From controlled experimental trial to = 2 everyday communication): How effective is intensive integrative therapy for stroke-induced chronic aphasia under routine clinical conditions? A study protocol for a randomized controlled trial

**DOI:** 10.1186/1745-6215-14-308

**Published:** 2013-09-23

**Authors:** Annette Baumgaertner, Tanja Grewe, Wolfram Ziegler, Agnes Floel, Luise Springer, Peter Martus, Caterina Breitenstein

**Affiliations:** 1 Faculty of Health and Social Sciences, Fresenius University of Applied Sciences, Alte Rabenstraße 2, 20148 Hamburg, Germany; 2 Department of Neurology, University of Muenster, Albert-Schweitzer-Campus 1, bldg A1, 48149 Muenster, Germany; 3 Faculty of Health and Social Sciences, Fresenius University of Applied Sciences, Limburger Straße 2, 65510 Idstein, Germany; 4 Entwicklungsgruppe Klinische Neuropsychologie (EKN), Dachauer Straße 164, 80992 München, Germany; 5 NeuroCure Clinical Research Center and Department of Neurology, Charité - Universitätsmedizin Berlin, Charitéplatz 1, 10117 Berlin, Germany; 6 Institute for Clinical Epidemiology and Applied Biometry, University of Tuebingen, Silcherstraße 5, 72076 Tübingen, Germany

**Keywords:** Aphasia, Chronic, Intensive therapy, Outcome, RCT, Recovery, Stroke, Treatment efficacy

## Abstract

**Background:**

Therapy guidelines recommend speech and language therapy (SLT) as the “gold standard” for aphasia treatment. Treatment intensity (i.e., ≥5 hours of SLT per week) is a key predictor of SLT outcome. The scientific evidence to support the efficacy of SLT is unsatisfactory to date given the lack of randomized controlled trials (RCT), particularly with respect to chronic aphasia (lasting for >6 months after initial stroke). This randomized waiting list-controlled multi-centre trial examines whether intensive integrative language therapy provided in routine in- and outpatient clinical settings is effective in improving everyday communication in chronic post-stroke aphasia.

**Methods/Design:**

Participants are men and women aged 18 to 70 years, at least 6 months post an ischemic or haemorrhagic stroke resulting in persisting language impairment (i.e., chronic aphasia); 220 patients will be screened for participation, with the goal of including at least 126 patients during the 26-month recruitment period. Basic language production and comprehension abilities need to be preserved (as assessed by the Aachen Aphasia Test).

Therapy consists of language-systematic and communicative-pragmatic exercises for at least 2 hours/day and at least 10 hours/week, plus at least 1 hour self-administered training per day, for at least three weeks. Contents of therapy are adapted to patients’ individual impairment profiles.

Prior to and immediately following the therapy/waiting period, patients’ individual language abilities are assessed via primary and secondary outcome measures. The primary (blinded) outcome measure is the A-scale (informational content, or 'understandability’, of the message) of the Amsterdam-Nijmegen Everyday Language Test (ANELT), a standardized measure of functional communication ability. Secondary (unblinded) outcome measures are language-systematic and communicative-pragmatic language screenings and questionnaires assessing life quality as viewed by the patient as well as a relative.

The primary analysis tests for differences between the therapy group and an untreated (waiting list) control group with respect to pre- versus post 3-week-therapy (or waiting period, respectively) scores on the ANELT A-scale. Statistical between-group comparisons of primary and secondary outcome measures will be conducted in intention-to-treat analyses.

Long-term stability of treatment effects will be assessed six months post intensive SLT (primary and secondary endpoints).

**Trial registration:**

Registered in ClinicalTrials.gov with the Identifier NCT01540383

## Background

Aging populations and higher survival rates in patients with acute stroke place increasing financial constraints on the health care system, requiring evidence-based interventions in stroke rehabilitation [1-3]. One of the most devastating conditions after stroke is aphasia, a disturbance in language function, which affects about 27% of all stroke patients [4]. About half of the initially affected patients still suffer from aphasia one year after stroke [5-7]. Apart from the emotional burden associated with aphasia [8], language dysfunction in the post-acute or chronic phase after a stroke is a major reason for failure of vocational rehabilitation [9]. Impaired communication ability commonly represents an obstacle to vocational and professional reintegration, thus incurring health care costs and losing potential contributors to the 'social contract’ (i.e., fiscal payments) [10].

Therapy guidelines recommend speech and language therapy (SLT) as the 'gold standard’ for aphasia treatment. Treatment intensity (i.e., ≥5 hours of SLT per week) appears to be a key predictor of SLT outcome [11]. The scientific evidence to support the efficacy of SLT is unsatisfactory to date [12] given the lack of randomized controlled trials (RCTs), particularly with respect to chronic aphasia (lasting for >6 months after the initial stroke). Even though recent evidence-based reviews support the efficacy of intensive aphasia therapy [2,13], the available evidence has not yet led to increased referrals for aphasia patients. This state of affairs may in part be due to a lack of high-quality RCTs with positive outcome as well as to a failure to administer a functional outcome measure. In the age of evidence-based medicine, however, there is a growing demand that SLT should be evidence-based and that outcomes should be operationalized with respect to participative gains [14,15].

Furthermore, motivated by the World Health Organization (WHO) guidelines regarding more patient-centred, outcome-oriented therapy schemes (International Classification of Functioning, Disability and Health [16]), aphasia rehabilitation facilities increasingly apply an integrative naturalistic therapy approach [17-19]. This integrative approach, which may be described as the agreed best practice, consists of both language-systematic and communicative-pragmatic exercises as well as a combination of one-on-one and group therapy settings. In addition, patients are often encouraged to supplement the gains of intensive therapy with self-administered computerized language exercises.

The efficacy of intensive SLT under such 'natural’ therapy conditions, however, has never been investigated in persons with chronic aphasia, although there is mounting evidence consistent with the assumption that SLT is efficacious when treatment intensity is sufficiently high. Specifically, aphasia therapy has been shown to be efficacious when treatment is provided for at least 5 hours per week for a period of several weeks [11]. A 2003 systematic review found that therapy studies which reported positive outcomes had administered an average of 8.8 hours of therapy per week [13]. The authors of this review noted, however, that only few of the studies included were rated to be of good quality; the majority of the studies were either of fair quality or did not fulfil basic quality criteria (such as randomizing participants to conditions). Since then, several empirical studies on SLT in post-acute/chronic aphasia have corroborated the assumption that short periods of intensive SLT (i.e., two weeks duration with several hours of SLT daily) significantly enhance linguistic functions even in the chronic stage after a stroke [20,21], with excellent long-term stability of therapy outcome [21,22]. However, the number of participants in these studies was relatively small; numbers ranged from 12 [21] to 28 [20] individuals with aphasia. Furthermore, apart from questionnaires asking patients and relatives to indicate the estimated level of communicative abilities, primary outcome measures in these studies were operationalized as changes in scores of standardized language test batteries. These types of outcome measures have been increasingly criticized because they do not reflect potential gains in everyday communicative language functions.

In the past two years, several RCTs with large numbers of participants have used primary outcome measures focused on functional communication ability [23-25]. In all of these trials, treatment started within the period of spontaneous remission after the initial stroke. Laska et al. initiated treatment sessions within two days after stroke, and administered 3.75 hours of therapy per week for a period of 21 days [25]. Therapy focused on ameliorating comprehension and naming abilities. After three weeks, no difference was found on the Amsterdam-Nijmegen Everyday Language Test (ANELT) between the treatment group and an untreated control group. Bowen et al. administered on average 18 hours total of agreed best practice of communication therapy over a period of up to four months (i.e., on average less than 2 hours per week) [23]. At six months post onset, no difference in a functional outcome measure was found between the treated group and a control group receiving social contact by contracted “visitors” with a similar frequency.

Compared to the significant improvements found in the studies administering intensive therapy with a frequency of at least 1 hour per day, the latter findings strongly suggest that the negative findings may partly have been due to the fact that therapy was administered with a frequency below the minimum of 5 hours per week recognized as being efficacious [13]. Thus, for patients with stroke-induced chronic aphasia, an evaluation of the efficacy of intensive integrative, systematic therapy with respect to everyday communication ability is urgently needed.

### Objective

This prospective randomized controlled endpoint-blinded trial aims to examine whether, in chronic aphasia, the integrative approach to intensive language and communication therapy translates into functional improvements of everyday communication as well as improved health-related quality of life.

The principal research question is two-fold: (i) how effective is intensive integrative (i.e. combined language-systematic and communicative-pragmatic) language therapy, as currently administered in German in- and outpatient aphasia rehabilitation facilities, in improving everyday communication in post-acute/chronic post-stroke aphasia; and (ii) to what degree are these improvements maintained over a period of 6 months?

## Methods/Design^a^

The current trial uses a prospective randomized open blinded end-point (PROBE) design [26] with a waiting-list control group. The trial was approved by the Ethics Committee of the Charité – Universitaetsmedizin Berlin, Germany (No. EA1/234/11, approval received in December 2011). In addition, the study has the approval of the local ethics committees of the respective German federal states in which the 17 participating neurorehabilitation centers are located (Table 1).

**Table 1:** List of participating institutions and contact person (i.e., Centre coordinator)

**Participating centre (location in italics)**	**Centre coordinator**
Max-Planck-Institut für Kognitions- und Neurowissenschaften *Leipzig* und Tagesklinik für Kognitive Neurologie (A. Villringer, H. Obrig, F. Regenbrecht)	Frank Regenbrecht
Brandenburg Klinik *Bernau* Waldfrieden (M. Jöbges, Maria Bley)	Maria Bley
Neurologische Klinik, Lehr- und Forschungsgebiet Neurolinguistik, Universitätsklinikum *Aachen* (C. Werner, K. Halm)	Katja Halm
Städtisches Klinikum München *Bogenhausen*, Klinik für Neuropsychologie (G. Goldenberg, R. Glindemann, W. Ziegler)	Ralf Glindemann
Aphasiestation der Neurologischen Klinik *Bad Aibling* am Standort Rosenheim ( E. König, F. Müller, G. Klingenberg)	Gudrun Klingenberg
St. Mauritius Therapieklinik *Meerbusch* (S. Knecht, B. Gröne)	Berthold Gröne
Median-Klinik *Grünheide*, Berlin (J. Knauss, K. Bölle, R. Baake)	Regina Baake
m&i Fachklinik *Enzensberg* (U. Steller, R. Sudhoff, K.-J.Schlenck)	Klaus-Jürgen Schlenck
m&i Fachklinik *Bad Liebenstein* (G. Pfeiffer, E. Schillikowski)	Eva Schillikowski
Aphasiezentrum Josef Bergmann, *Vechta* (F.-J. Ferneding, K. Billo, H. Hoffmann)	Kathrin Billo
Logopädisches und interdisziplinäres Behandlungszentrum für Intensivtherapie *Lindlar* (V. Middeldorf, S. Krüger, T. Keck)	Tina Keck
Asklepios Neurologische Klinik *Falkenstein* (K. Krakow, B. Wilde)	Barbara Wilde
Wicker-Klinik *Bad Homburg* v.d.H. (F. Reinhuber, C. Berghoff)	Carla Berghoff
Akademische Praxis für Sprachtherapie/Neurologische Praxis *Aschaffenburg* (I. Maser, W. Hofmann)	Ingeborg Maser
m&i Fachklinik *Herzogenaurach* (W. Schupp, C. Sous-Kulke)	Christa Sous-Kulke
Gräfliche Kliniken Moritz Klinik *Bad Klosterlausnitz* (D. Bätz, F. Hamzei)	Anke Oertel
MediClin Klinikum *Soltau* (A. Meyer, Katja Schulz)	Katja Schulz

### Study population

#### *Inclusion criteria*

• Non-haemorrhagic or haemorrhagic cortical, subcortical, or subcortico-cortical stroke;

• Presence of aphasia for at least 6 months;

• Age between 18 years and 70 years;

• German as (the first) native language;

• A score of at least 1 (between 0 and 5) on the communicative ability scale of the Aachen Aphasia Test/AAT [27];

• Less than the maximum score of 10 error points on the first of five subtests of the AAT Token Test (securing basic comprehension of spoken instructions).

In cases in which potential participants have been appointed a guardian, the guardian’s written approval to participation in the trial is required in addition to the signature of the patient. Further, if the patient has a guardian, the attending physician is asked to attest a patient’s ability to decide for him- or herself whether or not he or she wishes to participate.

#### *Exclusion criteria*

• No verifiable aphasia according to the criteria of the AAT;

• Aphasia due to traumatic brain injury or neurodegenerative diseases;

• Severe uncontrolled medical problems;

• Severe uncorrected-to-normal visual or auditory impairment;

• Participation in an alternate intensive intervention to relieve stroke symptoms during the past four weeks prior to enrolment.

### Therapy

Therapy consists of a combination of language-systematic and communicative-pragmatic approaches. In order to tailor therapy to the individual needs of the patients, we designed specific screening measures by which the individual severity of impairment across the various domains of language and communication may be identified in each patient prior to therapy. These screening measures examine language-systematic as well as communicative-pragmatic language functions. Individual therapy targets are set upon identification of performance levels in the screening measures. Contents of therapy are determined by a detailed standardized therapy manual which allows (i) establishment of a therapy plan tailored to the individual patient’s focus and level of language impairment(s); and (ii) adaptation of therapy material and/or therapy methods to the expected improvements in linguistic and communicative ability over the course of therapy^b^. Participants’ progress is monitored daily, and monitoring results as well as therapy content are documented by the attending therapists. All therapists in the participating centres underwent comprehensive training with the therapy manual prior to patient recruitment.

### Study design

**Figure 1:**
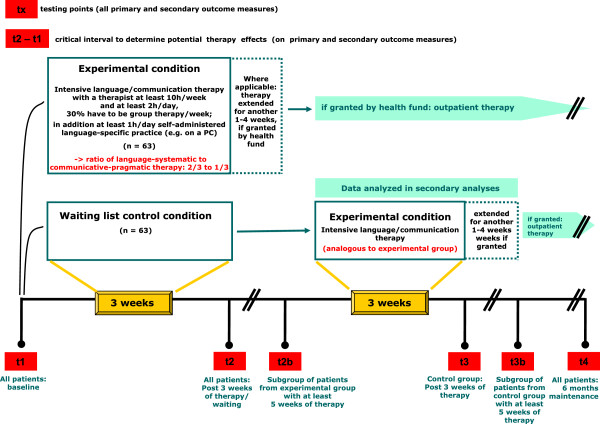
Trial design.

The study design is depicted in Figure 1. The inclusion of a waiting list control group, intended to control unspecific effects of language therapy, circumvents the ethical dilemma of withholding potentially efficacious treatment from patients. After a 3-week waiting period, the control group receives the same treatment as the experimental group.

A particular design aspect, namely the repeated test points t2b and t3b of Figure 1, is due to the heterogeneity of current therapy funding by German health funds. Initial funding is generally granted for 3 to 4 weeks, and may be extended for up to 7 weeks *during* the rehabilitation phase for some of the patients. For statistical reasons, we fixed a test point immediately after the 3-week intensive therapy (i.e., t2 and t3, respectively, in Figure 1) which includes *all* patients enrolled in the study. Whenever a patient’s individual funding period comprises at least 5 weeks of therapy, testing is repeated at the end of this patient’s total therapy period (i.e. t2b/t3b in Figure 1). The additional test point serves to measure further potential improvements beyond the initial 3-week therapy period. Initial screening, baseline testing, and follow-up tests are performed by specially trained 'study assessors’ (SAs), all with long-term experience in diagnosis and treatment of neurogenic speech and language disorders. All of the screening measures and tests are scored by the assessing SAs, and validated by an internal data review process, except for the primary outcome measure (see below). The primary outcome measure is evaluated by a specially trained endpoint committee (8 members) which is blinded with respect to patient group and time of assessment.

### Outcome measures

#### *Primary endpoint*

Mean difference on the ANELT [28] A-scale (understandability), operationalized as the difference in scores between the test point immediately after the 3-week intensive therapy (or waiting) period, and the test point immediately prior to the initiation of treatment (i.e., the difference of the scores, i.e., 't2’ minus those at test point 't1’; Figure 1). The ANELT tests verbal communicative ability in everyday communicative situations, shows a high concurrent validity with other standard linguistic assessment tools and is particularly suitable in the post-acute/chronic phase after stroke.

#### *Key secondary endpoint(s) (exploratory analysis)*

(i) Specially devised screening measures for language-systematic and communicative-pragmatic communication ability; (ii) the German version of the Stroke and Aphasia Quality of Life Scale-39/SAQOL-39 [29]; (iii) German Version of the Communicative Effectiveness Index/CETI [30]; (iv) B-scale (intelligibility) of the ANELT scenarios [28]; (v) ratings of the syntactic complexity of the ANELT scenarios using the AAT scoring system for spontaneous speech [27]; (vi) ratings of non-verbal communication skills on the ANELT scenarios (based on the Scenario test [31]).

To minimize practice effects due to repeated testing, the two parallel versions of the ANELT are alternated across testing sessions (retest reliability of the ANELT-I and ANELT-II: r >0.92 for the A-scale, r >0.74 for the B-scale [32]). Retest reliability of the other secondary outcome measures relating to quality of life is also satisfactory (CETI: r = 0.73, SAQOL-39: r = 0.98).

### Procedure

As part of the informed consent, patients are informed that the trial comprises an experimental condition and a waiting list (with a 3 week waiting period) condition. Patients who agree to participate are randomized to either the intensive therapy or the waiting list group, stratified according to centre, by a randomization procedure conducted in a central database by the Institute of Clinical Epidemiology and applied Biometry at the University of Tuebingen, Germany.

The outcome of the randomization procedure is communicated to the respective head of the SLT unit at each cooperating centre (Table 1) who is the only person to be informed about group allocation determined by the randomization procedure. Thus, the speech therapists conducting therapy are not informed about group allocation. Depending on their group assignment, patients either start therapy immediately (experimental group) or 3 weeks after the initial baseline session. The scoring of the primary endpoint (ratings on the ANELT A-scale) and the secondary ANELT endpoints is conducted independently from the therapists and study assessors performing the pre- and post-therapy assessments. That is, ANELT scoring is performed by an endpoint committee consisting of 8 raters, who are experienced in ANELT administration and scoring, and who have been specially trained to perform ANELT scoring in this trial. ANELT scorers are not involved in any other aspect of the trial in order to keep them blinded to group allocation (experimental, waiting list) and testing session (baseline or follow-up). Scoring of the ANELT is based on audio- and video-recordings collected by the study assessors. Each recording of an ANELT test is independently evaluated by two members of the endpoint committee; across the eight raters, the distribution of ANELT recordings for each patient follows a pseudo-randomized strategy.

### Power calculation

Sample size calculation was conducted for the comparison of mean change in ANELT A-scale score from t1 (baseline) to t2 (after 3 weeks of therapy or waiting) between the intensive language therapy group and the waiting list group (Figure 1). As a minimum difference of 8 points on the ANELT A-scale is considered clinically significant [32], group sample sizes of 63 were specified for each group in order to achieve 90% power to detect a difference of 10 points in the improvement of the mean ANELT A-scale score between the intensive treatment group after 3 weeks of intensive therapy and the waiting list group (difference of 1 point after 3 weeks of waiting), assuming a standard deviation for the difference in the ANELT A-scale score between t2 and t1 of 14 (10 for the waiting list group; Figure 1). Sample size was calculated for a two-sided Mann-Whitney test with significance level (alpha) of *P* <0.05. Assuming a patient non-participation rate of 25% prior to official study inclusion by randomization (for example due to the presence of exclusion criteria or no therapy funding by the respective sickness fund), 2 × 84 = 168 patients have to be recruited. The assumption of non-parametrical testing is rather conservative and the actual power will be larger if parametrical testing is possible.

### Statistical analyses

Analyses of primary and secondary endpoints will be performed in the (intention-to-treat) ITT population consisting of all randomized patients who received at least one day of therapy, or were at least one day on the waiting list. If data are normally distributed, parametrical methods will be used. The primary analysis will test for differences between the therapy group and the untreated (waiting list) control group with respect to the mean change (relative to baseline performance at the test point prior to the instantiation of therapy) on the ANELT A-scale at the test point immediately following the 3-week intensive therapy (or respective waiting) period. As opposed to the comparison group, which is not expected to demonstrate a clinically significant change between the two baseline assessments prior to SLT on the ANELT A-scale scores, the therapy group on average is expected to show a clinically significant improvement between the two test points. In the following paragraphs, statistical analyses are explained in more detail.

#### *Primary analysis*

The primary endpoint will be evaluated at baseline (t1) and immediately after 3 weeks of intensive therapy/waiting period (t2), by a statistical between-group comparison of the ANELT A-scale (ANCOVA) in an ITT design and with methods of multiple imputation taken into account. We expect that monotone missing data patterns will be observed (as described in [33]) and techniques appropriate for this situation will be applied. As a sensitivity analysis, the last-observation-carried-forward method will be applied.

Based on prior studies, we expect a therapy-induced improvement on the ANELT A-scale of M = 10 points (SD = 14) from baseline to immediately after 3 weeks of intensive therapy in the intensively treated group. For the waiting list group, we expect a change of M = 1 point (SD = 10) from baseline (t1) to the end of the 3-week waiting period (t2).

#### *Secondary analyses*

Performance on the ANELT A-scale will also be evaluated at baseline (t1) and immediately after 3 weeks of intensive therapy/waiting period (t2), by a statistical between-group comparison of the ANELT A-scale (ANCOVA) in a treated-per-protocol design. In patients who are granted an extension beyond 5 weeks of intensive language therapy by their respective sickness fund, performance on the ANELT A-scale from pre to post therapy will again be evaluated immediately after the total intensive therapy period (i.e., 5–7 weeks after the baseline assessment = t2b), by a statistical between-group comparison of the ANELT A-scale (ANCOVA) in an ITT design where the last observation will be carried forward. This design feature is required due to the current heterogeneous therapy funding practices by the sickness funds. Initial funding is generally granted for 3 weeks, and for some patients (about 20%) extended for up to 7 weeks. The decision about the extension is made *after* rehabilitation therapy has started. Therefore, total duration of therapy cannot be planned *a priori*. Secondary endpoints (language-systematic and communicative-pragmatic screening measures; German version of the SAQOL-39, German Version of the CETI, ANELT B-scale as well as syntactic and non-verbal communication ratings) will be analysed in analogy to the primary analysis at baseline (t1) and immediately after 3 weeks of intensive therapy/waiting period (t2), by a statistical between-group comparison (ANCOVA) in an ITT design. Where applicable, the secondary endpoints will again be evaluated after a variable therapy extension for up to 7 weeks total therapy (t2b) in a subgroup of patients (cf. secondary analysis of the ANELT A-scale). Maintenance of therapy gains will be evaluated 6 months after termination of the first 3 weeks of intensive therapy (t4, long-term outcome) by statistical comparisons with t1, respectively. The amount of outpatient therapy provided between t2/t2b and t4 will be statistically controlled by covariate analyses.

#### *Waiting list control group*

In analogy to the experimental group, therapy effects will be analysed immediately after 3 weeks of intensive therapy (t3). We expect a mean change of M = 10 after intensive therapy (comparisons of t1 and t3). We expect that therapy effects will be comparable to those of the experimental group. Outcome data of the waiting list group thus also serve to replicate the effect of intensive language therapy under routine clinical conditions. For control group patients who are granted more than 3 weeks of intensive language therapy, performance on the ANELT A-scale will again be evaluated immediately after the total period of therapy (i.e., 5–7 weeks of therapy, depending on the extension granted = t3b) in analogy to the experimental group. Again, we expect therapy effects to be comparable to those of the experimental group after 5–7 weeks of therapy. Maintenance of therapy gains will be evaluated 6 months after termination of the first 3 weeks of intensive therapy (t4, long-term outcome) by statistical comparisons with t1, respectively.

Taking into account that age, gender, time after stroke onset, aphasia type (fluent, non-fluent), aphasia severity (based on the AAT profile score), the total hours of therapy provided, type of stroke (cortical strokes with or without subcortical involvement), the amount of therapy-concomitant self-administered language practice (e.g. computer-aided practice), and medication and physio- and neuropsychological therapies might influence functional outcome, these factors will be included in a multivariate analysis with variable selection. No adjustment for multiplicity of testing will be provided. All analyses except for the primary analysis of the primary endpoint thus will not be strictly confirmatory.

## Trial status

The trial started in February 2012; patient recruitment started April 1, 2012. The last patient will be included on June 1, 2014 (last 6-month follow-up on January 31, 2015).

## Endnotes

^a^A written version of a talk about the study, held at the 2013 Annual Meeting of the German Society of Speech-, Language-, and Voice Pathology by CB, will be published as part of a national German conference proceeding in the journal 'Sprache-Stimme-Gehör’.

^b^The German therapy manual developed for FCET2EC is intended for publication (Eds.: Grewe, Baumgaertner & Springer†).

## Abbreviations

AAT: Aachen aphasia test; ANELT: Amsterdam-Nijmegen everyday language test; CETI: Communicative effectiveness index; SAQOL-39: Stroke and aphasia quality of life scale-39; SA: Study assessor; SLT: Speech and language therapy; ITT: intention-to-treat.

## Competing interests

The authors declare that they have no competing interests.

## Authors’ contributions

AB, CB, TG, AF, LS, PM, and WZ conceived the study and designed this trial. With input from all of the authors, AB submitted the proposal as principal investigator to the funding agency (German Federal Ministry of Education and Research [BMBF]). CB is the local trial coordinator. TG supervised the development of the therapy manual. AF is GCP certified neurologist, principal investigator at the Center for Stroke Research Berlin, and overseeing trial clinician for this study. LS was essential in developing therapy contents and the language-systematic screening measure. All authors read and approved the final manuscript.

## References

[B1] Teasell R Meyer MJ McClure A Pan C Murie-Fernandez M Foley N Salter K Stroke rehabilitation: an international perspective Top Stroke Rehabil 2009 16 44 56 10.1310/tsr1601-44 19443347

[B2] Cherney LR Patterson JP Raymer A Frymark T Schooling T Evidence-based systematic review: effects of intensity of treatment and constraint-induced language therapy for individuals with stroke-induced aphasia J Speech Lang Hear Res 2008 51 1282 1299 An updated version of the original review dated October 2010 is available at http://www.asha.org/uploadedFiles/EBSR-Updated-CILT.pdf 10.1044/1092-4388(2008/07-0206) 18812489

[B3] Salter K Teasell R Bhogal S Zettler L Foley N Aphasia. Evidence-Based Review of Stroke Rehabilitation 2012 15 http://www.ebrsr.com/papers_details.php?3

[B4] Lesniak M Bak T Czepiel W Seniow J Czlonkowska A Frequency and prognostic value of cognitive disorders in stroke patients Dement Geriatr Cogn Disord 2008 26 356 363 10.1159/000162262 18852488

[B5] Laska AC Hellblom A Murray V Kahan T Von Arbin M Aphasia in acute stroke and relation to outcome J Intern Med 2001 249 413 422 10.1046/j.1365-2796.2001.00812.x 11350565

[B6] Pedersen PM Vinter K Olsen TS Aphasia after stroke: type, severity and prognosis. The Copenhagen aphasia study Cerebrovasc Dis 2004 17 35 43 14530636 10.1159/000073896

[B7] Ferro JM Mariano G Madureira S Recovery from aphasia and neglect Cerebrovasc Dis 1999 9 Suppl 5 6 22 10473916 10.1159/000047571

[B8] Hilari K Northcott S Roy P Marshall J Wiggins RD Chataway J Ames D Psychological distress after stroke and aphasia: the first six months Clin Rehabil 2010 24 181 190 10.1177/0269215509346090 20103578

[B9] Hofgren C Bjorkdahl A Esbjornsson E Sunnerhagen KS Recovery after stroke: cognition, ADL function and return to work Acta Neurol Scand 2007 115 73 80 17212608 10.1111/j.1600-0404.2006.00768.x

[B10] Kolominsky-Rabas PL Heuschmann PU Marschall D Emmert M Baltzer N Neundörfer B Schöffski O Krobot KJ Lifetime cost of ischemic stroke in Germany: results and national projections from a population-based stroke registry: the Erlangen stroke project Stroke 2006 37 1179 1183 10.1161/01.STR.0000217450.21310.90 16574918

[B11] Cherney LR Aphasia treatment: intensity, dose parameters, and script training Int J Speech Lang Pathol 2012 14 424 431 10.3109/17549507.2012.686629 22731660 PMC3698219

[B12] Brady MC Kelly H Godwin J Enderby P Speech and language therapy for aphasia following stroke Cochrane Database Syst Rev 2012 5 CD000425 10.1002/14651858.CD000425.pub3 22592672

[B13] Bhogal SK Teasell R Speechley M Intensity of aphasia therapy, impact on recovery Stroke 2003 34 987 993 10.1161/01.STR.0000062343.64383.D0 12649521

[B14] Frattali CM Measuring Outcomes in Speech-Language Pathology 1998 New York: Thieme

[B15] Dollaghan CA The Handbook for Evidence-Based Practice in Communication Disorders 2007 Baltimore: Brookes Publishing Co.

[B16] World Health Organization (WHO) International Classification of Functioning, Disability and Health 2001 http://www.who.int/classifications/icf/en/

[B17] Springer L Stemmer B, Whitaker HA Therapeutic approaches in aphasia rehabilitation Handbook of the Neuroscience of Language 2008 1 San Diego: Elsevier 397 406

[B18] Byng S Integrating therapies Adv in Speech-Lang Pathol 2001 3 67 71 10.3109/14417040109003713

[B19] Moriz M Geißler M Grewe T Grötzbach H, Iven C ICF in der Stationären Aphasietherapie ICF in der Sprachtherapie [ICF in Speech Therapy] 2009 Idstein: Schulz-Kirchner Verlag 39 59

[B20] Meinzer M Elbert T Wienbruch C Djundja D Barthel G Rockstroh B Intensive language training enhances brain plasticity in chronic aphasia BMC Biol 2004 2 20 10.1186/1741-7007-2-20 15331014 PMC515310

[B21] Barthel G Meinzer M Djundja D Rockstroh B Intensive language therapy in chronic aphasia: which aspects contribute most? Aphasiology 2008 22 408 421 10.1080/02687030701415880

[B22] Meinzer M Djundja D Barthel G Elbert T Rockstroh B Long-term stability of improved language functions in chronic aphasia after constraint-induced aphasia therapy Stroke 2005 36 1462 1466 10.1161/01.STR.0000169941.29831.2a 15947279

[B23] Bowen A Bowen A Hesketh A Patchick E Young A Davies L Vail A Long AF Watkins C Wilkinson M Pearl G Ralph MA Tyrrell P Effectiveness of enhanced communication therapy in the first four months after stroke for aphasia and dysarthria: a randomised controlled trial BMJ 2012 345 e4407 10.1136/bmj.e4407 22797843 PMC3396461

[B24] de Jong-Hagelstein M van de Sandt-Koenderman WME Prins ND Dippel DWJ Koudstaal PJ Visch-Brink EG Efficacy of early cognitive–linguistic treatment and communicative treatment in aphasia after stroke: a randomised controlled trial (RATS-2) J Neurol Neurosurg Psychiatry 2011 82 399 404 10.1136/jnnp.2010.210559 20935327

[B25] Laska AC Kahan T Hellbolm A Murray V von Arbin M A randomized controlled trial on very early speech and language therapy in acute stroke patients with aphasia Cerebrovasc Dis 2011 1 66 74 10.1159/000329835 PMC3343759 22566984

[B26] Hansson L Hedner T Dahlöf B Prospective randomized open blinded end-point (PROBE) study. A novel design for intervention trials Blood Press 1992 1 113 119 10.3109/08037059209077502 1366259

[B27] Huber W Poeck K Weniger D Willmes K Aachener Aphasie Test 1983 Göttingen: Hogrefe

[B28] Blomert L Kean ML Koster C Schokker J Amsterdam Nijmegen everyday language test construction, reliability and validity Aphasiology 1994 8 381 407 10.1080/02687039408248666

[B29] Hilari K Byng S Lamping DL Smith SC Stroke and aphasia quality of life scale-39 (SAQOL-39): evaluation of acceptability, reliability, and validity Stroke 2003 34 1944 1950 10.1161/01.STR.0000081987.46660.ED 12855827

[B30] Lomas J Pickard L Bester S Elbard H Finlayson A Zoghaib C The communicative effectiveness index: development and psychometric evaluation of a functional communication measure for adult aphasia J Speech Hear Disord 1989 54 113 124 2464719 10.1044/jshd.5401.113

[B31] van der Meulen I van de Sandt-Koenderman WM Duivenvoorden HJ Ribbers GM Measuring verbal and non-verbal communication in aphasia: reliability, validity, and sensitivity to change of the scenario test Int J Lang Commun Disord 2010 45 424 435 10.3109/13682820903111952 20144004

[B32] Blomert L Koster C Amsterdam-Nijmegen Test Voor Alledaagse Taalvaardigheden - Handleiding 2008 Amsterdam, The Netherlands: Hogrefe

[B33] van Buuren S Flexible Imputation of Missing Data 2012 Boca Raton, USA: CRC Press, Francis & Taylor Group

